# Hospital and Institutional News

**Published:** 1916-05-20

**Authors:** 


### HOSPITAL AND INSTITUTIONAL NEWS.

### SIR PERCEVAL NAIRNE AND HIS PORTRAIT.

The many friends of Sir Perceval Nairne and the *Dreadnought* Seamen's Hospital will be interested to know that an excellent portrait of him in oils by Mr. George Henry, A.R.A., is hung on the line in the First Gallery at the Royal Academy, Number 14. In many ways the portrait is an excellent one, and it is further remarkable from the uniform appearance given to the morning frock-coat which Sir Perceval is wearing. The portrait is to hang in the board-room of the *Dreadnought* Hospital, and a replica will be presented to Sir Perceval Nairne in due course. A small committee, of which Sir Patrick Manson, G.C.M.G., F.R.S., is chairman, raised a fund for the painting and presentation of this portrait, and a list of the contributors is in preparation, each one of whom will receive a photogravure of it. Any surplus will be handed over to the Samaritan Fund of the Seamen's Hospital. Few men are better beloved or more highly and deservedly respected than Sir Perceval Nairne, and the list of subscribers cannot fail to have a genuine interest for all hospital workers. A memoir of Sir Perceval Nairne appeared in The Hospital of April 22, 1911, page 93.

### THE BISHOP'S LAST.

In a Note in these columns last week dealing with a letter signed by three bishops and many others, urging all citizens to forego meat and alcohol for at least one day a week—Thursday and Monday—we expressed the view that it would have been better had each signatory stuck to his last. The *Sussex Daily News*, which seems to have strong vegetarian sympathies, in an article on our comments, describes the latter point as confusion of metaphor. Does our contemporary forget the story of the bishop who, when travelling, was worried by a talkative gentleman of the road, who described his methods of business, and demanded to know what line the bishop travelled in? The bishop's answer was, "I travel in souls." Or, again, we might remind our contemporary that a last is a wooden model of a portion of anatomy which enables the skilled artist to protect and beautify an important part of the human body. The object of the bishops, too, was to beautify the whole body, no doubt, and if their ambition was to succeed, it must rest upon perfected models for both bodies and souls. It will be seen, therefore, that our metaphor not only has the support of custom and practice, but might claim to rest upon authorities.

### NERVOUS BREAKDOWN IN THE NAVY.

It is announced in the *Morning Post* that a scheme is being prepared for dealing with cases of nervous breakdown in the Navy. It is hoped to establish a small hospital for officers of the Navy and its allied branches who may be suffering from acute nervous breakdown due to the war, and to obtain for petty officers and men similarly affected suitable treatment apart altogether from lunacy administration: to do for the Navy, in fact, what has already been done for the Army. It is also hoped to establish a pioneer hostel for petty officers and men who have recovered from acute breakdown but are not yet fit to resume the ordinary occupations of life. The intention is that this hostel shall be self-supporting; the scheme, when completed, will be submitted for approval to the Admiralty, which has already expressed itself as sympathetic to such a proposal.

### WITTENBERG'S HEROIC DOCTORS.

In The Hospital of April 29, 1916, page 95, we expressed grave dissatisfaction at the inadequacy of the recognition in the shape of honours made to the doctors of the R.A.M.C. who worked so heroically and continuously at Wittenberg when typhus and other diseases decimated the prisoners there. We are glad therefore to record that the Royal College of Physicians, Edinburgh, has taken action by offering diplomas of membership to Major H. E. Priestley, Captain A. C. Vidal, and Captain J. L. Lauder. We hope that the greater universities and medical and surgical colleges may be moved to take similar action as one way of intimating the dissatisfaction of the profession with the inadequate honours so far conferred in this case.

### THREE NEW BENEVOLENT SOCIETIES.

It has been suggested, and the suggestion has been encouraged officially, that benevolent societies should be formed for the benefit of the families of officers and other ranks of the Medical Services auxiliary to the regular Royal Army Medical Corps—namely, the Special Reserve, the Territorial Force, and the new Armies. The Director-General is to preside at a meeting which will be held on June 1 to consider the question. There are certain obvious difficulties in the idea. One is that it is quite impossible to learn the general consensus of opinion among those concerned, either officers or men, for the vast majority of them are abroad. Another is that the administration of any such societies will necessarily have to be left in the hands of either senior War Office officers or of London consultants; for no one else is certain of remaining in or near London for any length of time. Another is that it is only during the war that such societies will be needed; for Territorials, Special Reserve, and most or all the temporarily commissioned or enlisted will be civilians again as soon as war is over, and therefore within the scope of civilian schemes and agencies.

### A "RARA AVIS": THE MEDICAL "C.O."

Conscientious objectors are, happily, few and far between in the ranks of the medical profession, and it is certainly unusual to find a doctor whose views carry him to the extent of refusing even to lend a hand in the succour of the wounded. At the Argyllshire Appeal Tribunal last week Dr. J. C. MacCallum, tuberculosis officer of the county, appealed for exemption as a conscientious objector. The local Tribunal had decided that Dr. MacCallum had not made out a case for exemption, and suggested that there was nothing to prevent him showing a true Christian spirit by placing his medical skill at the disposal of his wounded fellow-countrymen. In his appeal Dr. MacCallum said his father was a parish minister who had preached consistently the essential sinfulness of war, and the family had for many years been conspicuously anti-militarist in its activities. Current events had done nothing to shake applicant's ingrained repugnance to war. On the contrary, the spectacle of the never-ending contest which was drenching Europe in blood had but served, he said, to steel his resolution against what was shown clearly to be a dastardly crime against humanity. Further, he was unable to realise that there was any logical difference in morality between combatant and non-combatant service. The appeal was dismissed.

### ECONOMY IN THE ANNUAL REPORT.

In our correspondence column will be found a letter from the treasurer and the chairman of Charing Cross Hospital explaining that the annual report of this institution will not be sent out broadcast to subscribers as usual, though a small number will be printed and supplied to those subscribers who write to ask for it. By this measure of war economy a saving of somewhere between two and three hundred pounds will be effected. The idea may be commended to the notice of other hospital authorities; and there is one direction, at least, in which it could be extended with advantage. At some institutions it is customary to print as part of the annual report a complete list of all subscriptions and donations ever since the foundation; and this section, which of course grows more unwieldy every year may come in time to constitute three-quarters, or even nine-tenths, of the whole report. The need for war economy warrants the suspension of a custom which, when confined to living beneficiaries, has the advantage of the maintenance of an up-to-date and complete list of those genuinely interested in the charity. Such a list is at its best the backbone of financial success when there are special needs to be met. Its profitable and effective use entails the presence of an able chairman or secretary, or both.

### SURCHARGING OF STAFFORDSHIRE PANEL PRACTITIONERS.

The surcharging of panel practitioners for excessive or extravagant prescribing is an unpleasant necessity in certain cases, and probably the Insurance Committees detest having to do it as much as the medical profession hates hearing of its occurrence. Many committees have been forced to carry out this duty, though the sum total of the medical men who incur such penalties is very small compared with the numbers on the panels. A striking instance of the contrariness of those who get themselves thus penalised is furnished by a case recently before the Staffordshire Insurance Committee. A panel doctor in that county ordered prescriptions during 1915 which cost the committee £359. It was estimated that the requirements of the patients could have been adequately met for less than half this sum, £175. The doctor would have had no one to blame but himself if he had been surcharged the difference, £184. However, he was let off with a surcharge of £75, enough perhaps to make him think twice about his future course of conduct. As an example of his prodigality it was stated that for two of his prescriptions, added together, the cost of the active drugs was threepence, while the cost of flavouring ingredients was one shilling and fivepence.

### INVITING EPIDEMICS TO LONDON.

Dr. C. Halford Badcock, of Croxted Road, Norwood, has written to the Lambeth Borough Council drawing attention to the serious increase in the amount of illness during the last two or three months in his district, which he states "is due mostly to non-notifiable diseases, and of a kind due to dust, and generally prevalent in August. From my own experience," he adds, "I feel sure that there is more illness in this specified area than at any time during the last ten years, due to dust. The conditions of the road have been almost intolerable." His complaint is supported by a number of residents in the vicinity. The road has only lately been used by motor-'bus traffic to Norwood and the Crystal Palace, and, as it was not originally constructed for heavy traffic, it has been badly cut up. In other parts of London, especially along the main thoroughfares where the motor-'bus traffic is heavy, the same complaints have arisen. Borough Councils in several Metropolitan districts, with a view to keeping down expenses, have curtailed the amounts they usually spend on road repair and street sweeping and cleansing. But from Norwood's experience it is manifest that the curtailment or scamping of hygienic precautions in the way of street and road sweeping and repairs may become a scandal. If such folly is persisted in, it may inflict upon London an epidemic, or possibly the return of typhus or plague outbreaks before the war is out. Where are the medical officers of health for the county and districts of London? Let them do their duty by prompt action or resign their posts.

### SANATORIUM STARS.

Dr. Melville Rees, senior medical officer at Winsley Sanatorium, has been fined £1 for a contravention of the Lights Restriction Order. A constable, in giving evidence, declared that on the evening in question he saw seven bright lights showing from the Winsley Sanatorium. After watching them for a quarter of an hour four of them were extinguished, and five minutes later, the remaining three being still visible, he proceeded to the institution and interviewed one of the medical staff. The lights, which he described as showing up almost like stars, could be seen ten miles away owing to the high position of the sanatorium. Dr. Rees said that all windows were shaded, that the patients had received instructions, and that he made tours of inspection himself. The

104 patients included many children, who might perhaps have pulled up the blinds. All lights were put out at 9 P.M. A police sergeant said that he realised the difficulties at the institution, but the prominence of the sanatorium made every precaution important. The Bench, as stated, only imposed a small fine.

### A CURIOUS POINT IN SANITARY LAW.

Some little time ago a Middlesbrough woman was charged before the local Stipendiary with allowing a person to come in contact with the body of a child recently dead of an infectious disease (measles). The Stipendiary dismissed the case upon a point of law, which seems to have been that the person allowed to run the risk of infection did not actually touch the body, and therefore did not come into "contact" with it. The King's Bench, on appeal, overruled the Stipendiary, thus establishing that actual physical contact is not essential in the construction of the statute, but that so near an approach as to incur risk of infection is all that need be proved. The Stipendiary, to whom the case was referred back, accordingly had to convict, but refused to impose any penalty. Inasmuch as the medical officers of health for Middlesbrough and for Darlington, who both gave evidence, emphasised the necessity for impressing on the public the great importance of taking measles seriously, and of reducing its incidence as far as possible, it is a little disheartening to find their efforts counteracted by a Stipendiary who is evidently not alive to the urgency of the question, and does his best to nullify and obstruct their educational endeavours.

### THE TREATMENT OF ADENOIDS AT LIVERPOOL.

At a recent meeting of the Liverpool City Council there was some opposition, which was pressed to a division but defeated by a four-fifths majority, to a recommendation of the Education Committee for the establishment of a clinic for the treatment of tonsils and adenoids among children in the elementary schools. Part of the opposition was based upon grounds of economy, and upon the shortage of medical men caused by the war: the ability and willingness of voluntary hospitals to do the work was also a matter of divergent opinions. But the principal opponent of the measure relied first on the contention that this work is not properly that of an education authority at all; and, secondly, on the supposition that the "oppression and dragooning of the parents" will necessarily be involved in the working of the clinic, in spite of the express statement in the resolution that this is not intended. It is quite probable that the war and our recent education in methods of government have been sufficiently an object-lesson to prevent this latter point from being taken very seriously. If it is true, as suggested, that Liverpool parents care so little for their children's health that they will obstinately refuse (on "conscientious" grounds, presumably) to allow them to be relieved of the serious dangers of enlarged tonsils and adenoids, then the sooner they are "dragooned" a bit the better for their children and the future of the State. But we think better of them.

### THE MEDICAL EXAMINATION OF RECRUITS.

A Yorkshire medical man, Dr. F. H. Squire, the medical officer of health for Pudsey, has handed over his fees for examining men offering to enlist under the Derby Scheme to one of the principal workers in the local recruiting movement, with a request that it may be given to what that gentleman considers the most useful objects. Two working parties for soldiers and the Mayoress's fund have been chosen. Perhaps the most unfortunate feature of the whole system of medical examination for recruits has been the extent to which official instructions to medical men have been varied; so that the practitioner has rarely felt free to act solely upon the physical condition which his examination has revealed to him. The result has been grievances on every side. Medical men have been subjected to criticism on the ground that no general standard has been maintained; many recruits have complained of injustice, when the real cause of the trouble has been the constant change of attitude on the part of the authorities. In view of all this Dr. Squire's gift has been much appreciated locally.

### SOME TIT-BITS IN A HOSPITAL "GAZETTE."

The good things in the May number of the *Gazette* of the 3rd London General Hospital contain one or two genuine titbits. One is the matron's really admirable account of "a memorable afternoon" spent by her and her charges at Buckingham Palace. At the end of tea and entertainments, Miss Holden writes, "When I had time to look round, I saw to my horror that our men had raided the stage, and were wearing great bouquets of flowers in their hats and button-holes, and many of them had captured the 'reserved tickets' put on special seats. I felt at least that I should be sent to the Tower for such behaviour, and was quite thankful to get out without being asked any questions. However, no one seemed to mind." Admirable candour! We all sadly know the ghastly "correct" and gruesome manner in which such occasions are usually not described, but made so tedious that one wonders whether everyone really did laugh at this "happy speech," or applaud that gracious condescension as we are told they did. Miss Holden knows a trick worth two of that: to say what actually occurred—not merely what was on the programme. The result is that her article is capital reading throughout.

### THE SCULPTURE OF FACES IN PLASTICINE.

Two more good things are the account of the hospital's new chicken-run (under "orderly" management) and Lieut.-Col. Porter's note on the establishment of the new department for modelling faces in plasticine for those who have suffered injury. Details were given in The Hospital of April 15 last, page 43. Without Derwent Wood, the sculptor, the work could not have been done. That is enough to justify our preoccupation with the art of this month's literary contents of the *Gazette*. The illustrations are excellent, especially the little insets by a "probationer" Vera Down, Miss de la Touche. Read the editorial claims for this hospital as the home of many successful experiments, and then doubt, if you can, that a staff of artists (which fortune gave it) is not a guarantee of ability; hence the chance for a good magazine which will attract the able contributors among the patients to it. The staff now, by the way, includes a composer, Private Vidal.

### THE "SOUTHERN CROSS" IN MAY.

The editor of the *Southern Cross*, the journal of the 1st Southern General Hospital, Birmingham, records the staff's regret at the recent departure of 103 N.C.O.'s and men who had been trained there. The quality of his journal, however, has not suffered. It has improved. "N. R." has a real ear for verse (see stanzas 5, 7, and the last two of his poem "Forgotten"), and the best of lines have that mysterious perfume which (deliberately to mix the metaphor) makes a line that *sings*. The "Who's Zoo" cartoon this month is worthy of its predecessors, and if, as we believe, cartoons should be blossoms on the stalk of a general idea, it would have been difficult to invent a wittier one. As to the cartoons, another "Finance" is a sort of Watts and Raemaekers combined. With a crown on his head and a heavy chain and collar round his neck, the Colossus, sitting on the ground, holds the Crown Prince of Prussia (last of the Hohenzollerns) and (is it?) his papa one in each hand. If it is meant to represent the economic strength of the Allies, the artist's identification of such strength with the typical figure of Mammon has perhaps more suggestiveness than most observers will be equal to. A remarkable illustration of an old carved "doctor's sign-board" is well worth reproducing, and provides an interesting instance of the earlier forms of professional advertising. It is dated 1623, and has a beauty of its own.

### WHO EDITS WAR HOSPITAL GAZETTES?

In the last number of the *Craigleith Hospital Chronicle* is recorded the death of its first editor. In the memoir of its late editorial chief we learn that "he" was a lady, Nurse A. F., Paulin. "When the work of the hospital started, and it was seen how valuable it would have been to have the help of voluntary workers, the first person appealed to for such work was Miss Paulin." When the idea for a hospital magazine was duly conceived, Miss Paulin was again approached, and again successfully. Her efforts as editor, we are told, "were unceasing to secure contributions and advertisements." Her monument is that share of the series of the *Craigleith Hospital Chronicle* which has appeared so successfully under her editorship, for we gather that she did not act as editor all the time. This raises the question as to the prevalence of the nurse as editor of the war-hospital magazines.

### THE NURSE SUB-EDITOR.

When a woman writes well, like Florence Nightingale, for example, there is something more than arrangement and concentration in her writing. There is a neatness, a thinness of style in good women writers which makes their sentences come off the reel like silk. Men's sentences generally come off like rope. It is a difference in kind. In addition, many women seem to possess the sub-editorial gift; and probably the best edited war-hospital magazines are those in which, while the editor may be a man, the sub-editor is a woman. The Florence Nightingales of this world, who possess both the editorial and the sub-editorial gifts (which are quite different, though all gifts lie somewhere between the two), by sheer force of ability will be driven to exercise the more responsible and, as things are, the scarcer one alone. No one has time for both. But the experience seems to show that some women have a specific gift for sub-editing.

### 1 MAN 150 VOTES.

Whatever be the result of the special meeting called to decide as to the continuance of the absolute and scandalous system of cumulative voting in connection with the Blackpool Victoria Hospital—the grotesqueness of the situation does not, as some people seem to think, redeem its gravity. By the traditional abuse of monopoly six men control nearly 1,000 votes; and as the former system of collusion showed at the time of the old scandal two years ago, this caucus can overwhelm every other interest, and can combine to constitute its own board by a system of sham "election," which includes all the pretensions of representation with all the corruption of a job. In fact a job lot is exactly what such a procedure creates on the board. It was devised to create them. It did create them. That is the evil which has to be eradicated. We have often prophesied that, in places where a hospital has lost all touch with modern standards and modern ideas, the day will come for a strike of patients. At Blackpool the former medical board *did* strike. If the evil which at bottom roused (though it did not occasion) that old dispute is allowed to persist by any "amending" of the trust deed without abolishing the caucus vote, then a strike of subscribers may follow. If it does not, and the public is still long suffering, then we predict that one day a strike of patients will. That is the end to which the Blackpool Victoria Hospital is steering with the complacency of a pilot who has gone to sleep.

### LAMP DAY.

The people of this country, and of London especially, have evidently not yet got tired of those forms of street collection which consist of the sale of flags, badges, or other insignia by bevies of damsels posted at street corners and similar points of pedestrian congestion. Every conceivable war charity, and even a normal peace-time charity, such as the Lifeboat Institution, has its "day," and more or less remuneratively holds up to ransom all who use the streets. One of the most successful of recent "days" was that which was held on the anniversary of Miss Nightingale's birthday, when little yellow pasteboard lamps were sold in vast quantities in the streets. On this occasion three enterprises particularly connected with women's efforts were the beneficiaries, though probably not one in ten of those who decorated their coats with lamps knew what any of the three are or what they aim at. The three organisations are the Women's Service Bureau, the Women's Emergency Corps, and the Star and Garter Fund. Our own view is that these incessant "days" are becoming a nuisance, and that public opinion will react against them to their total extinction; but this may be a mistaken prophecy, for certainly there are few symptoms of any such reaction at present.

### AN UNFORTUNATE DAY AT DUDLEY.

The Guest Hospital, Dudley, seems to have had a bad day on May 2, for two patients died there under anæsthetics on that date. The report of only one inquest has reached us, though we presume that both cases were inquired into by a Coroner's jury. In this case the patient was an oldish man suffering from acute peritonitis due to the rupture of one of the hollow viscera: the sort of patient, that is, who might come to grief in the hands of the most skilled anæsthetist. The urgency of his condition rendered it necessary that any risks should be run, as the sole chance of saving him lay in immediate operation. The anæsthetist was a junior house surgeon, and possibly the emergency character of the operation precluded the summoning of a more experienced administrator. The doctor who performed the *post-mortem* examination thought that the anæsthetic had contributed towards the fatality, and suggested that in view of the other death on the same day an analysis of the anæsthetic used should be carried out. This suggestion is not of much practical utility: it would be more interesting to know whether the administrator in the second case was or was not a skilled anæsthetist.

### SOLDIERS AND THE DRUG HABIT.

The Order made by the Army Council, under the Defence of the Realm Regulations, prohibiting the sale of certain drugs to any member of His Majesty's Forces, comes as no surprise. On more than one occasion allusion has been made in The Hospital to the inadequacy of the Pharmacy Acts to prevent soldiers from freely obtaining supplies of such drugs as cocaine and morphine, and in view of the evidence that the "drug habit" is not non-existent in the Army, it was clearly in the interests of the State to put a stop to the practice and to make it an offence for any person, whether a hawker of pernicious drugs or a properly qualified pharmacist, to supply a demand which clearly ought not to be catered for. The Schedule of Drugs which may not be supplied to soldiers is a comprehensive one, comprising as it does practically all the narcotic and hypnotic substances for which any request is likely to be made. To prevent any interference with the *bona-fide* use of these drugs for medical purposes the Order contains a provision enabling any of the scheduled substances to be obtained on the written order of a medical practitioner provided the prescription is dated and signed with his full name and qualifications, and marked with the words "Not to be Repeated." It is unfortunate that no power exists to extend these regulations to the whole population.

### PRESCRIPTIONS FOR HOSPITAL OUT-PATIENTS.

The great advance in price of many drugs and chemicals is adding greatly to the expenditure of hospitals, and a new system of dealing with the medicine required by out-patients is to be tried at the Birmingham General Hospital. Instead of the hospital supplying free medicine as formerly, it is proposed to give a prescription to those out-patients requiring medicines—this will be made up by a local chemist and charged on the basis of the "tariff of charges" paid for National Health Insurance prescriptions—the charge to be paid by the patient. The tendency to waste or imbibe medicine in excess will, it is hoped, be checked by this method, and, incidentally, the hospital funds will be relieved of a heavy expenditure. It will be interesting to follow the course of this proposed arrangement, for the procedure is not without its disadvantages. If the foregoing arrangements do not prove satisfactory, an arrangement might be made for out-patients to pay "cost price" for medicines supplied from the hospital dispensary, though this would probably meet with opposition from pharmaceutical bodies.

### FALSE ECONOMIES.

The Local Government Board has administered a somewhat severe rebuke to the Health Committee of the Stockport Corporation, in consequence of the refusal of the Corporation to make further provision at present for maternity and child welfare work. After expressing regret at this decision, the L.G.B. observes that "the importance of conserving the infant life of the population renders it desirable that steps to extend the work should be taken, even at the present time when strict economy is required in the expenditure both of public bodies and of private individuals. It is especially important [the communication adds] that there should be adequate arrangements for the home visitation of children under school age and of expectant mothers, and it is quite clear that the Council's present staff is inadequate for the needs of the borough in this respect." After this piece of official advice perhaps the Stockport Authority will decide to set its house in order and resist "economies" which only mean imperilling the health and welfare of the future generation.

### THE WAY TO DEAL WITH A CRITIC.

Criticism is often met by the assertion that the critic is incapable of doing the work he criticises. Logically, the retort involves a fallacy easy of demonstration. The ability to detect a fault does not necessarily carry with it the power to remedy or prevent it—a fact every golf-player or driver of a motor-car knows. Alderman Dawson has recently criticised very severely the financial methods of the Birkenhead Board of Guardians. In revenge, the Guardians have co-opted him a member of the board. Future developments will be awaited with interest. Probably both the Alderman and the board will be the gainers. A man with Alderman Dawson's knowledge and energy is bound to exert a powerful influence upon the board's policy, and at the same time he will learn much from personal experience of Poor-Law problems and administration.

### THE POOR-LAW AND DISCHARGED SOLDIERS.

At the last meeting of the Conway Board of Guardians a discussion arose on the treatment of two discharged soldiers who had become chargeable to the Union. In the one case a soldier who had become insane had been sent to an asylum as a pauper patient; in the other a wounded soldier suffering from epilepsy had been sent from the local hospital to the Poor-Law infirmary. It was decided to prepare a case to be put before the National Poor-Law Conference with a view to a united appeal to the War Office on behalf of soldiers discharged as unfit for service, who without Government assistance would become chargeable as paupers. Cases similar to those at Conway have occurred in other Unions, and are likely to occur with increasing frequency the longer the war lasts. The conditions under which soldiers are invalided from the Service vary greatly. The discharged soldier may or may not be entitled to a pension; he may be invalided as the result of wounds or illness directly due to military service, or his incapacity may have nothing to do with it. The greatest sympathy of the public will naturally be with those whose incapacity has been contracted as a result of active service. In the case of many of these hospital treatment of some kind may be required for years, and the problem of its provision should be faced at once. The public will not be satisfied that they should be handed over to the present Poor-Law authorities. The State should provide the institutions necessary, and the most economical way in which it could do so would be for it to acquire some of the existing Poor-law buildings and utilise them for the purpose.

### A REDUCTION OF SALARY AND A PROTEST.

At the last meeting of the Driffield Board of Guardians the following letter from Dr. Brand, the medical officer of the workhouse, was read: "From newspaper reports I find that at your meeting of April 20 you passed a resolution, recommended by your committee, to reduce my salary as medical officer of the Children's Homes by one-half. I protest against your decision, and shall be glad to be informed why I alone of the various officers concerned should be singled out for reduction of salary, since the work is equally curtailed in each case. I beg to point out that not only is the reduction of 50 per cent. not in proportion to the alleged reduction of work, which is in all probability only temporary, but that an annual salary of £5 is not a fair remuneration for the work still to be done by me as a professional man. I also regret to have to complain of the extreme discourtesy you have shown me in giving no intimation either of your intention to reduce my salary, or of your having passed a resolution to that effect, a discourtesy of which I am glad to believe that any other body of men but yourselves is incapable. It is fortunate for me, however, that you do not possess the power, as you appear to have the will, to reduce my salary, and I think it only fair to inform you that I have appealed to a higher tribunal, from whom I expect to have at least fair treatment." Dr. Brand's complaint of the discourtesy with which he has been treated is unanswerable, and several members of the Board expressed their regret that he had not been consulted. The resolution of the Board is inoperative until confirmed by the Local Government Board, and it is very improbable that that Board will adopt the view that the services of the medical officer of a Children's Home are adequately rewarded by an honorarium of £5 per annum.

### THIS WEEK'S DRUG MARKET.

The downward tendency of the prices of some of those drugs which, until recently, had been continually moving upwards in value is still noticeable. It is true that this tendency is not very pronounced, but nevertheless there is no doubt that it exists, and buyers of drugs for hospitals should take particular pains to inquire about the market conditions of all drugs that have to be purchased at the present time, for the market is in a critical condition. The considerable increase both in the British and the American output of some of the synthetic drugs is one of the causes of the lull in the upward movement of prices; and another reason is the fact that agents who have been buying for Russian account have found it necessary to sell some of their purchases because of the difficulty that has arisen in shipping goods, other than war supplies, to Archangel. Both citric and tartaric acids are slightly cheaper. Bromides can be bought at something below makers' reduced prices. The value of cocaine is by no means so firmly maintained. Salicylate of soda and salicylic acid can be purchased at a little below the quotations recently ruling. Potassium permanganate is again rather lower in price. On the other hand, phenacetin is somewhat dearer in consequence of the fact that the Swiss Government has placed an embargo on its export. Phenolphthalein is difficult to obtain. Guaicol carbonate is scarcer than ever. Sugar of milk is again dearer.

### TO OUR READERS.

**Contributions are specially invited from any of our readers to these columns. They should deal with topical subjects and news. They must be authenticated for the information of the Editor only. The minimum payment if published is 5s. There is no hard-and-fast rule as to space, but notes of about twenty lines in length are preferred.**

